# Protective Effects of Crocin Against Methotrexate-Induced Hepatotoxicity in Adult Male Albino Rats: Histological, Immunohistochemical, and Biochemical Study

**DOI:** 10.7759/cureus.34468

**Published:** 2023-01-31

**Authors:** Ghada AbdelKader, Eman Z Abdelaziz, Ranya Hassan, Sahar M Greish, Noha M Abogresha, Basma O Sultan, Einas M Yousef, Shereen Morsi

**Affiliations:** 1 Department of Human Anatomy and Embryology, Faculty of Medicine, Suez Canal University, Ismailia, EGY; 2 Department of Pharmacology, Faculty of Medicine, Suez Canal University, Ismailia, EGY; 3 Department of Clinical Pathology, Faculty of Medicine, Suez Canal University, Ismailia, EGY; 4 Department of Medical Physiology, Faculty of Medicine, Suez Canal University, Ismailia, EGY; 5 Department of Physiology, School of Medicine, Badr University in Cairo (BUC), Cairo, EGY; 6 Department of Internal Medicine, Faculty of Medicine, Suez Canal University, Ismailia, EGY; 7 Department of Histology and Cell Biology, Faculty of Medicine, Menoufia University, Shibin El Kom, EGY

**Keywords:** methotrexate-induced hepatotoxicity, oxidative stress, methotrexate, hepatotoxicity, crocin, apoptosis

## Abstract

Background: Among the many known adverse effects of methotrexate (MTX), hepatotoxicity stands out as a major drawback that limits its therapeutic applicability. There is growing evidence that crocin has antioxidant, anti-hyperglycemic, cardioprotective, and anti-inflammatory effects. This study's aim is to evaluate the potential protective effect of crocin against MTX-induced liver damage in rats using biochemical, histological, and immunohistochemical analyses.

Methods: Twenty-four adult male albino rats were split into four groups at random (six rats/group) as follows: normal control (saline, intraperitoneal (i.p.) injections), crocin-treated (100 mg/kg daily for 14 days, i.p.), MTX-treated (20 mg/kg single i.p. injection on day 15), and crocin/MTX-treated groups (crocin 100 mg/kg/day for 14 days, i.p. + MTX 20 mg/kg single i.p. injection on day 15). On day 16 of the experiment, blood and tissue specimens were used to assess the liver functions, oxidative stress markers, transforming growth factor beta 1 (*TGF-β1*), caspase-3, *BCL-2*-associated X protein (*BAX*), and B-cell lymphoma 2 (*BCL-2*) expression.

Results: The results of the current research revealed the protective actions of crocin against MTX-induced hepatotoxicity. Our results showed that crocin possesses antioxidants (decrease malondialdehyde (MDA), increase glutathione (GSH) levels, and enhance catalase (CAT) and superoxide dismutase (SOD) enzymatic activity), anti-fibrotic (decrease *TGF-β1*), and anti-apoptotic (decrease *BAX* and *caspase-3* expression while increase *BCL-2*) actions in liver. Moreover, crocin administration along with MTX restores the normal histological structure of hepatic tissues.

Conclusion: The data presented in the current study using an in vivo animal model support the notion that crocin should be further studied in humans to assess its potential hepatoprotective effects against MTX-induced liver damage.

## Introduction

Methotrexate (MTX) is an anti-folic acid medication and an aminopterin stable derivative that inhibits deoxyribonucleic acid (DNA) synthesis and repair. MTX is a very potent cytotoxic drug that alters cellular metabolism and so suppresses cell growth [1]. It was initially prescribed for children with acute leukemia, and it was later used in the treatment of psoriasis and rheumatoid arthritis. The MTX-cytotoxic effect, however, is not limited to cancer cells and affects many other normal tissues, including the stomach mucosa, gall bladder, hematopoietic cells of the bone marrow, and liver [2].

Hepatotoxicity is one of the key recognized toxicity profiles of MTX, which limits its therapeutic applicability [3]. The hepatotoxic mechanism of MTX is yet unclear, nevertheless, conceivable explanations might be proposed [1,4]. One possible mechanism of MTX-induced hepatotoxicity is its action on the intestinal mucosa, which disrupts intestinal barrier functions, allowing bacteria to translocate to the liver and cause hepatotoxicity [5]. Furthermore, MTX-enhanced intestinal permeability has been associated with hepatic inflammation, increased hepatic transaminases, and abnormal liver histological architecture such as increased liver fibrosis, cirrhosis, and hepatocyte apoptosis [5]. It has previously been reported that MTX's cytotoxic action on the liver is mediated in part by its ability to inhibit the conversion of homocysteine to methionine, resulting in an increase in homocysteine, which damages the endoplasmic reticulum and stimulates fat accumulation in hepatocytes, the proinflammatory cytokines, and hepatic stellate cells, all of which lead to liver fibrosis [4].

With adequate monitoring and medication regimens of MTX, the frequency of high transaminases has decreased to 22% in the past few years [6]. However, around 5% of patients receiving continuous low-dose MTX might develop advanced hepatic fibrosis or cirrhosis. MTX prescription, even at modest doses, is contraindicated in individuals with risk factors such as pre-existing liver disease, alcohol usage, obesity, and diabetes mellitus [3]. Many adjuvants with cytoprotective properties, such as ursodeoxycholic acid and carotene, have been recommended for users to reduce the occurrence of MTX-induced adverse effects [4,6]. Likewise, saffron, a perennial stemless plant used as a food additive, has recently been the focus of many research studies because of its recognized pharmacological characteristics like anti-inflammatory, anti-cancer, anti-hyperlipidemic, and cardioprotective effects [7-12]. The primary active ingredient in saffron is picrocrocin, along with its derivatives such as safranal, flavonoid compounds, and crocin.

Crocin is an active ingredient in saffron that is responsible for its red color [12,13]. Crocin is water soluble and heat stable, and is composed of gentiobiose and crocetin, which are disaccharides and carboxylic acids, respectively. A growing body of evidence showed that crocin has antitumor, antioxidant, anti-hyperglycemic, cardioprotective, anti-inflammatory, and DNA-protective effects [14,15]. Crocin pretreatment protects the stomach mucosa from ischemia-reperfusion damage by increasing messenger ribonucleic acid (mRNA) expression and the activity of certain antioxidant enzymes [16]. Although crocin has been demonstrated to have hepatoprotective properties against oxidative stress, other putative mechanisms involved in safeguarding the liver from MTX-induced damage have been less investigated. Hence, this research was carried out to examine the potential protective effect of crocin against MTX-induced liver damage in rats, putting a focus on the antioxidant and antiapoptotic effects. To accomplish this aim, liver functions, oxidative stress markers, transforming growth factor beta 1 (*TGF-β1*), caspase-3, *BCL-2*-associated X protein (*BAX*), and B-cell lymphoma 2 (*BCL-2*) expression were all assessed in a rat model.

## Materials and methods

Animals

Twenty-four male albino rats (120-150 g) were employed in the current experiment. The rats were acquired from the Ophthalmology Research Institute in Giza, Egypt. They were housed in standard rat cages and allowed a week to acclimate before the experiment began. They were maintained on a regular chow diet, water, and reversed cycles of darkness and light. All the guidelines of the Ethics Committee, Faculty of Medicine, Suez Canal University were followed during the experiment (Research# 4331). Every attempt was made to minimize the total number of animals utilized and their anguish.

Experimental procedure

Rats were randomly designated into four groups (six rats/group) as follows: group I (normal control group): rats were given intraperitoneal (i.p.) injections of an equal volume of normal physiological saline (vehicle) for two weeks; group II (crocin-treated group): crocin (Sigma-Aldrich, London, UK) dissolved in saline was administered to rats (100 mg/kg/day, i.p.) once daily started on the first day of the experiment and continued for 14 days [17]; group III (MTX-treated group): on day 15 of the experiment, a single dose of MTX (20 mg/kg, i.p., with normal saline as solvent) was administered to the rats [17]; group IV (crocin/MTX-treated group): starting from the first day of the study, the rats were given crocin (100 mg/kg/day, i.p.) for 14 days followed by a single MTX injection (20 mg/kg, i.p.) on day 15.

On day 16, a day following MTX injection, by i.p. injection of 50 mg/kg of ketamine and 5 mg/kg of xylazine, rats were anesthetized [18]. Blood samples were drawn from the abdominal aorta and left to stand for four hours at ambient temperature before being spun in a centrifuge to separate the serum from the blood cells, which were then used for various biochemical assays. For the tissue-based biochemical and histopathological analyses, samples of the liver from the right lobe were taken.

Serum biochemical analyses

Liver Function Tests

To assess liver function, the serum activities of alanine aminotransferase (ALT), aspartate aminotransferase (AST), alkaline phosphatase (ALP), and lactate dehydrogenase (LDH) were measured in all blood samples. These enzymes were tested by the enzymatic assay kits for colorimetry (Bio-diagnostics, Giza, Egypt) utilizing a spectrophotometer for ultraviolet (UV) visibility (UV-1601-PC; Shimadzu, Kyoto, Japan) [19]. Total protein and serum albumin were also measured.

C-Reactive Protein (CRP)

The serum level of CRP was quantified using automated COBAS 6000 (module 501, Roche Diagnostics, Basel, Switzerland) according to the manufacturer's guidelines. Every sample was carried out in duplicate.

Assessment of oxidative stress markers

In all experimental groups, liver tissues were minced and homogenized. In ice-cold saline, a homogenate of 10% w/v was prepared. The prepared homogenates were then centrifuged for 15 minutes at 18,000g (14°C). The supernatants of liver homogenate were collected to assess hepatic malondialdehyde (MDA) level as a lipid peroxidation indicator using the colorimetric assay kits (catalog # MD 2529, Bio-diagnostics) according to the manufacturer's recommendations. Levels of glutathione (GSH), as well as activity of catalase (CAT) and superoxide dismutase (SOD), were all assessed as indicators of liver antioxidant status using the colorimetric assay kits (Bio-diagnostics, catalog # GR 2511, CA 2517, and SD 2521, respectively).

Quantitative real-time polymerase chain reaction (qRT-PCR) of *TGF-β1*

*TGF-β1 *expression levels were determined using qRT-PCR. Frozen liver tissues from each rat were used to extract the total ribonucleic acid (RNA) of different study groups following the manufacturer's directions with the Qiagen tissue extraction kit (Qiagen, Germantown, Maryland). Spectrophotometry was used to assess the retrieved RNA's purity and concentration (dual-wavelength spectrophotometer, Beckman, Irvine, California). The isolated RNA was utilized in the preparation of complementary DNA (cDNA) considering the manufacturer's instructions with a high-fidelity reverse transcription kit (Fermentas, Waltham, Massachusetts). Using the Applied Biosystems apparatus and StepOne^TM^ software version 3.1 (Thermo Fisher Scientific, Waltham, Massachusetts), the reaction was incubated at 95°C for 10 minutes, followed by 40 cycles of 95°C for 15 seconds and 60°C for one minute. The *TGF-β1* levels were standardized to glyceraldehyde 3-phosphate dehydrogenase (GAPDH) mRNA levels in each sample [19]. The relative gene expression was calculated using the 2−^ΔΔ^CT method in comparison to the controls [20]. The *TGF-β1* primer sequences used were as follows: sense: 5'-TTGCCCTCTACAA CCAACACAA - 3'; and antisense: 5'-GCTTGCGACCCACGTAGT A-3'. All experiments were performed in triplicate.

Histopathological study

The liver samples were taken and preserved for 24 hours in 10% neutral buffered formalin. The liver samples were then washed, dehydrated in ascending alcoholic gradients, and paraffin-embedded for preparation of paraffin wax blocks. Sections of 4 um thickness were prepared and processed for hematoxylin and eosin (H&E) staining, as detailed by Bancroft et al. [20]. To overall assess the severity of hepatic tissue lesions in different study groups, a semiquantitative scoring method was employed as described by Ragab et al. [21]. Five high-power fields from each animal were examined by two independent histopathologists. A score ranging from 0 to 3 was used, with 0 indicating no histopathological alterations, 1+ denoting pathological changes in less than 20% of the studied fields, 2+ indicating histopathologic changes in between 20% and 60% of the investigated fields, and 3+ indicating histopathologic changes in more than 60% of the fields examined.

Immunohistochemical (IHC) study and morphometric analysis

To determine the transcription of *BAX* and caspase-3, and *BCL-2* proteins in the liver sections, IHC staining was carried out in accordance with Torlakovic et al.'s protocol [22]. The liver sections from various experimental groups were incubated with monoclonal antibodies of *BAX*, *BCL-2*, and caspase-3 (Dako, Carpinteria, California) at a dilution of 1:200. *BAX*, *BCL-2*, and caspase-3 were considered positive in cells that exhibited brown precipitate. Negative control was prepared by incubating the tissue specimens with the antibody diluent while omitting the primary antibody. The IHC profiler plugin in the ImageJ software (National Institutes of Health, Bethesda, Maryland) has been used to assess the expression of three markers [23]. Using the 3,30-diaminobenzidine (DAB)-stained cytoplasmic option of the IHC plugin, 10 non-overlapping fields were assessed from each experimental group using immune-stained sections (200x).

Statistical analysis

The collected data were analyzed using the Statistical Package for Social Sciences (SPSS, version 22, IBM Corp., Armonk, NY), which were given as mean ± standard deviation (SD). The difference in mean values among groups was calculated using the one-way analysis of variance (ANOVA), which was then confirmed by the Bonferroni multiple comparison test. Two-tailed p-values were given, and a p < 0.05 was considered to be significant. Every possible comparison was conducted between the study groups.

## Results

Biochemical results

The present data showed that liver functions were significantly deteriorated with MTX administration. As depicted in Table 1, rats treated with a single injection of MTX demonstrated a highly significant increase (p < 0.001) in the serum levels of AST, ALT, and ALP enzymes in comparison to control and crocin-treated groups. On the other hand, administration of crocin before MTX injection significantly reduced the AST level when compared with the MTX-treated group. Although, both ALT and ALP levels were reduced in rats treated with crocin and MTX combined, no significant difference was detected when compared to the MTX-treated group. It is worth noting that rats treated with MTX had non-significant differences in LDH, serum albumin, and total protein levels when compared to the normal control group (Table 1).

**Table 1:** Liver function tests among studied groups Data are presented as mean ± SD. ^1^ ANOVA test and Bonferroni test.^ 2^ Kruskal-Wallis test and adjusted Bonferroni test. ^#^ Compared to the normal group at p < 0.05. ^@^ Compared to the crocin-treated group at p < 0.05. ^$^ Compared to the MTX group at p < 0.05. AST: aspartate aminotransferase; ALT: alanine aminotransferase; ALP: alkaline phosphatase; LDH: lactate dehydrogenase, MTX: methotrexate.

Liver function	Normal control	Crocin	MTX	Crocin/MTX	P-value
AST	156.8 ± 38.2	173.7 ± 69.4	322.7 ± 15^#@^	237 ± 25.3^#$^	<0.001^1^
ALT	24.3 ± 0.8	29 ± 10.6	88.3 ± 2.6^#@^	77.5 ± 8.2^#@^	<0.001^2^
ALP	77.7 ± 12.9	93.8 ± 41.5	500.8 ± 8.1^#@^	326.33 ± 7.9^#@^	<0.001^2^
LDH	1209.7 ± 45.7	1716.7 ± 593.8	1921 ± 83.9	1795.5 ± 1005.4	0.079^2^
Serum albumin	3.4 ± 0.37	3.1 ± 0.19	3.1 ± 0.25	3.1 ± 0.33	0.162^1^
Total protein	7.6 ± 0.5	7.4 ± 0.9	6.8 ± 0.4	7 ± 0.3	0.145^1^

**Figure 1:**
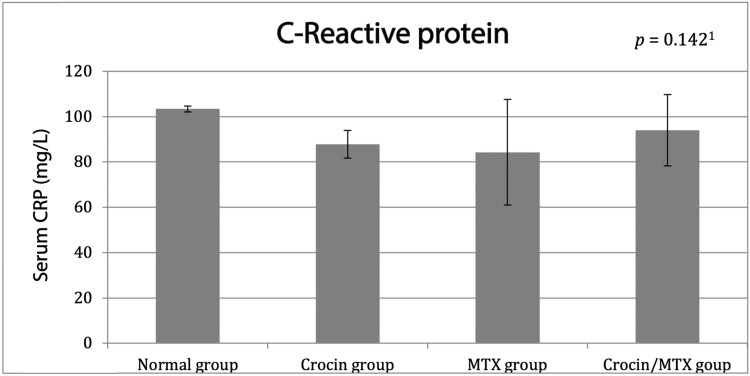
CRP level among studied groups Data are expressed as mean ± SD and were analyzed using ^1^one-way ANOVA and Bonferroni post-hoc test (n = 6 for each group). MTX: methotrexate: CRP: C-reactive protein.

Next, the liver inflammation status in all experimental groups was evaluated using the CRP, a marker of inflammation. Nonetheless, as seen in Figure 1, no statistically significant difference could be detected between the research groups.

Markers of lipid peroxidation and oxidative stress

In this study, our results revealed that a single injection of MTX significantly raised (p < 0.001) the lipid peroxidation marker (MDA) level in liver homogenates when compared to the normal control and the crocin-treated groups. Furthermore, when comparing the group treated with MTX to both the control and the crocin-treated groups, our results revealed a significant reduction (p < 0.001) of GSH level and decreased SOD and CAT enzymatic activity in liver tissue homogenates. Pretreatment with crocin in the crocin/MTX-treated group mitigated the impact of MTX on lipid peroxidation and oxidative stress markers. Crocin administration in rats injected with MTX reduced MDA while increasing GSH levels and enhancing the activity of CAT and SOD in liver tissues, as demonstrated in Table 2.

**Table 2:** Oxidative stress markers among studied groups Data are presented as mean ± SD. ^1^ ANOVA test and Bonferroni test. ^2^ Kruskal-Wallis test and adjusted Bonferroni test. ^#^ Compared to the normal group at p < 0.05. ^@^ Compared to the crocin-treated group at p < 0.05. ^$^ Compared to the MTX group at p < 0.05. MDA: malondialdehyde; GSH: glutathione; CAT: catalase; SOD: superoxide dismutase; MTX: methotrexate.

Oxidative stress markers	Normal control	Crocin	MTX	Crocin/MTX	P-value
MDA (nmol/g tissue)	0.26 ± 0.04	0.36 ± 0.18	0.90 ± 0.33^#@^	0.51 ± 0.02^$^	<0.001^1^
GSH (μmol/g tissue)	0.239 ± 0.026	0.238 ± 0.023	0.078 ± 0.003^#@^	0.138 ± 0.023^#@$^	<0.001^1^
CAT (μ/g tissue)	1.08 ± 0.051	1.07 ± 0.009	0.5 ± 0.011^#@^	1.03 ± 0.005^#@$^	<0.002^1^
SOD (μ/g tissue)	0.037 ± 0.051	0.034 ± 0.003	0.014 ± 0.003^#@^	0.027 ± 0.006^$^	<0.001^2^

TGF-β1 mRNA expression in liver tissue using qRT-PCR

**Figure 2:**
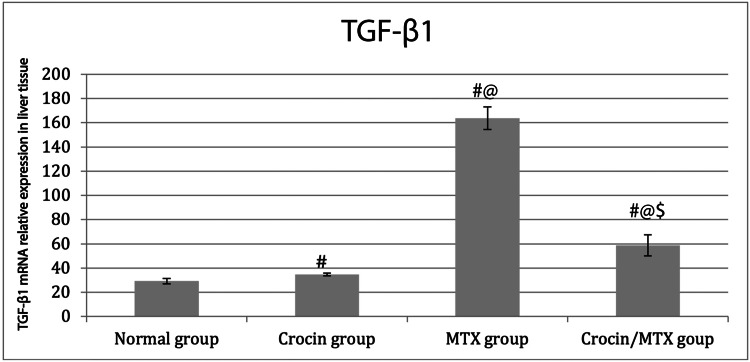
TGF-β1 mRNA relative expression level among studied groups using qRT-PCR Data are expressed as mean ± SD and were analyzed using one-way ANOVA and Bonferroni post-hoc test. ^#^ Compared to the normal group at p < 0.05. ^@^ Compared to the crocin group at p < 0.05. ^$^ Compared to the MTX-treated group at p < 0.05 (n = 6 for each group). TGF-β1: transforming growth factor beta 1; MTX: methotrexate; qRT-PCR: quantitative real-time polymerase chain reaction.

Administration of MTX induced a marked increase in the mRNA expression level of *TGF-β1*, a profibrogenic marker, when compared with both control and crocin-treated groups (p < 0.05). However, the administration of crocin together with MTX induced a significant downregulation of *TGF-β1* when compared with the MTX-treated group. However, *TGF-β1* expression remained significantly higher among rats treated with crocin/MTX in comparison to that of the normal control group (Figure 2).

Histopathological results

**Figure 3:**
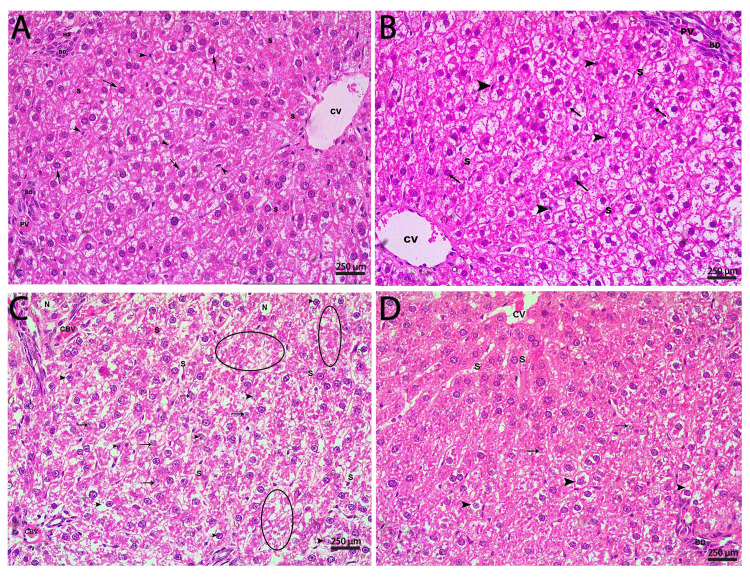
Sections in the rat liver stained with H&E from different study groups (A) Normal control group with normal histological structure of the liver showing a central vein (CV), a branch of the hepatic artery (HA), a branch of the portal vein (PV), a bile duct (BD), sinusoids (S), hepatocytes (arrow), and von Kupffer cells (arrowheads). (B) Crocin-treated group showing a central vein (CV), a branch of the portal vein (PV), a bile duct (BD), sinusoids (S), hepatocytes (arrow), and von Kupffer cells (arrowheads). (C) MTX-treated group showing congested blood vessels (CBV), dilated and congested sinusoids (S), hepatocytes in different stages of necrosis (arrow), hydropic degeneration (arrowheads), areas of cellular necrosis (circles), and necrotic foci (N). (D) Crocin/MTX-treated group with no congested blood vessels (CV), fewer dilated sinusoids (S), fewer necrotic hepatocytes (arrow), and fewer hepatocytes with hydropic degeneration (arrowheads) (H&E 400x). H&E: hematoxylin and eosin; MTX: methotrexate.

The histological framework of liver tissue in both the normal control and crocin-treated groups was comparable (Figures 3A, 3B). H&E-stained sections of the liver from both groups demonstrated the classical view of the liver lobules, with the central vein in the core and the portal triad branches at the lobules' periphery. The central vein and peripherally located portal triad (hepatic artery, portal vein, and bile duct) were connected with columns of radially distributed hepatocytes with prominent, centrally placed, and spherical nuclei. The single-cell width hepatocytic columns were separated by blood sinusoids with scattered Kupffer cells.

Sections from the MTX-treated group stained with H&E disclosed congestion of blood vessels with dilated and congested sinusoids, which indicates hepatoportal vascular congestion. Hepatocytes demonstrated cytoplasmic vacuolation, hydropic degeneration, and variable stages of necrosis, including nuclear fading, shrinkage, and fragmentation. Whole areas of cellular absence were present with variable-sized areas of necrotic foci (Figure 3C). Notably, there was no cholestasis or inflammatory cellular infiltration in the group receiving MTX (Table 3). Crocin administration in combination with MTX, on the other hand, improved the histological architecture of the liver tissue in comparison to the MTX-treated group. Based on the histopathological assessment and no vascular congestion, few hepatocytes (<20%) demonstrated necrotic or hydropic degenerative changes (Figure 3D and Table 3). These findings underline the crocin's hepatoprotective effects against MTX-induced liver damage.

**Table 3:** Semiquantitative scoring of H&E-stained sections of various histopathological parameters in different experimental groups H&E: hematoxylin and eosin; MTX: methotrexate.

Group parameters	Normal control	Crocin	MTX	Crocin/MTX
Hepatoportal and sinusoidal congestion	--	--	+++	--
Cloudy swelling and hydropic degeneration	--	--	+++	+
Cellular necrosis (nuclear pyknosis, karyorrhexis, and karyolysis)	--	--	+++	+
Inflammatory cellular infiltrate	--	--	--	--
Cholestasis	--	--	--	--

Immunohistochemical and morphometric results

**Figure 4:**
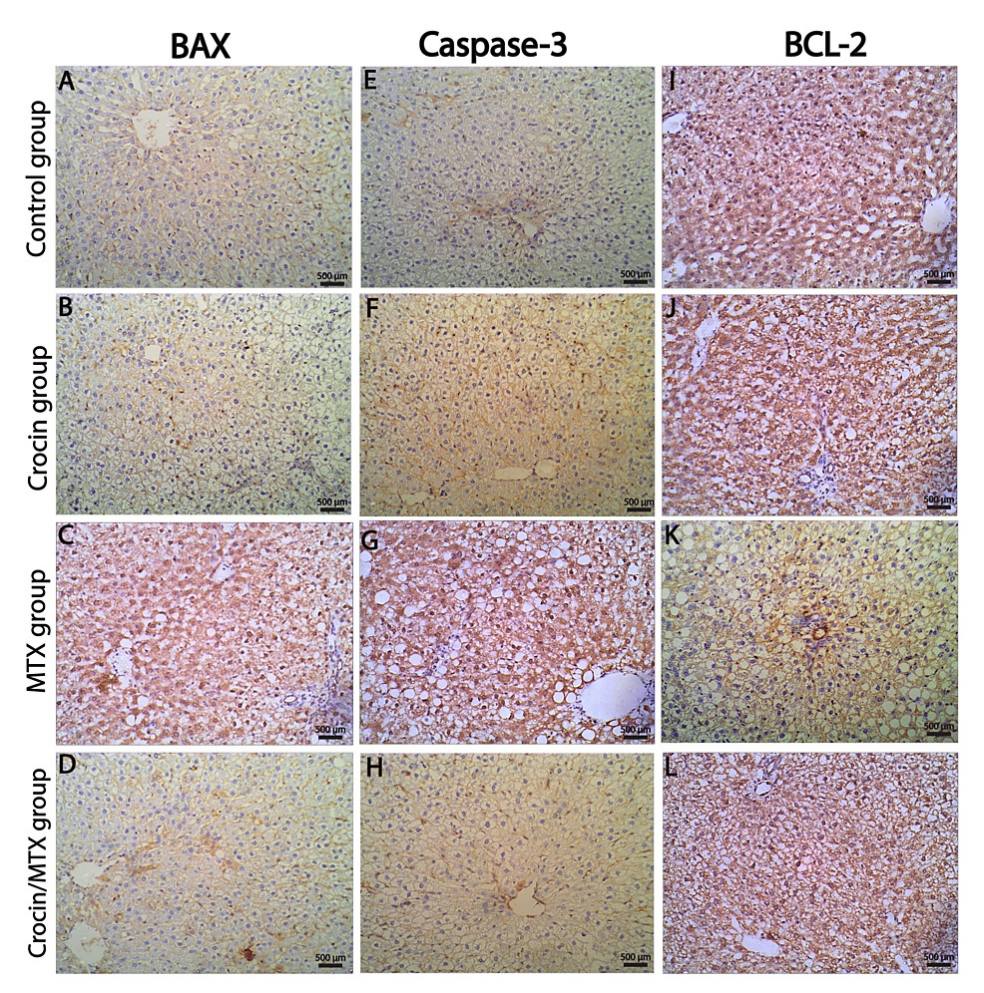
Liver immunostaining for BAX (A-D), caspase-3 (E-H), and BCL-2 (I-L) in hepatic tissue of all studied groups (immunostaining 200x) BCL-2: B-cell lymphoma 2; BAX: BCL-2-associated X protein; MTX: methotrexate.

**Figure 5:**
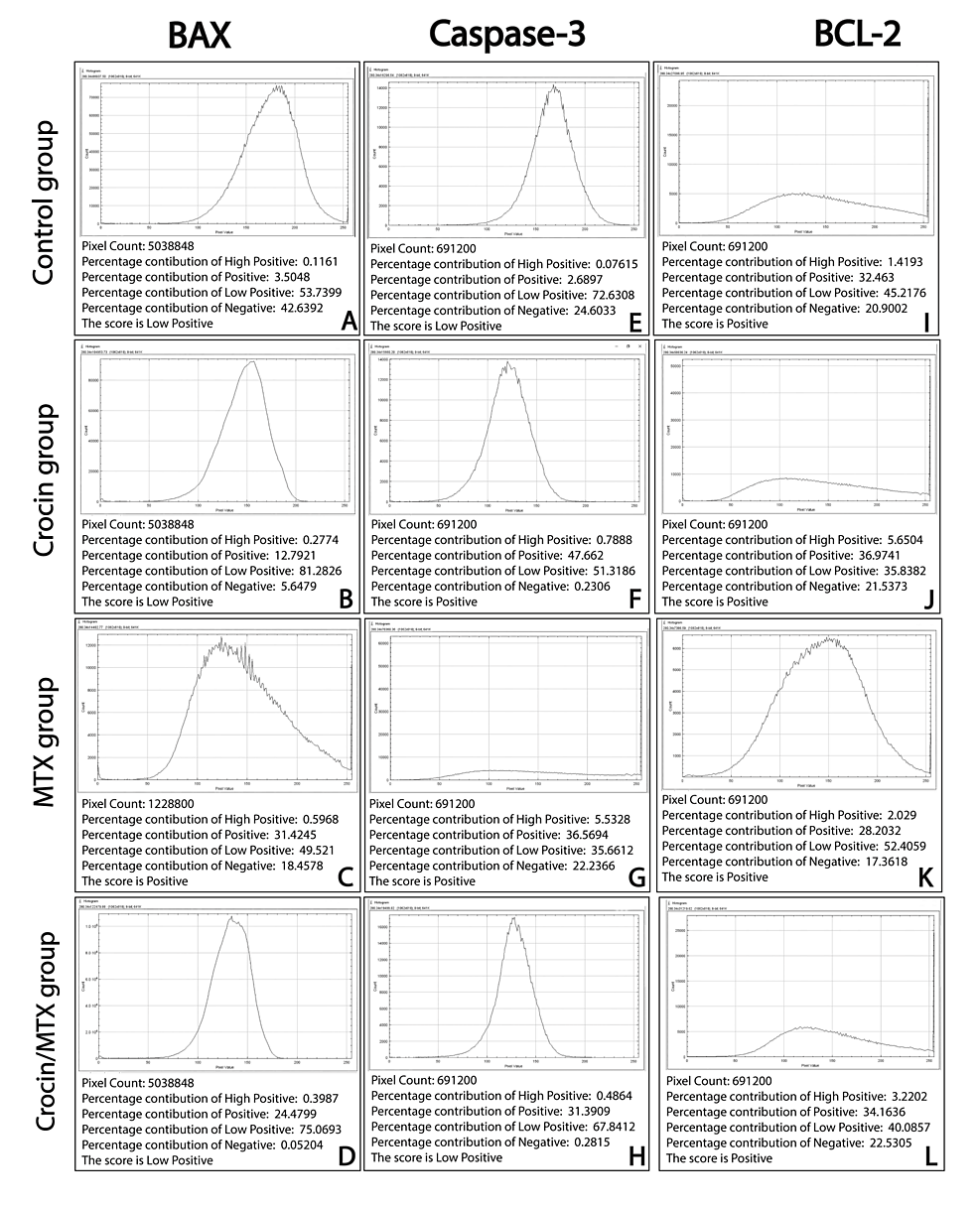
Histogram profiles of IHC scoring of BAX (A-D), caspase-3 (E-H), and BCL-2 (I-L) created by the ImageJ program (IHC profiler plugin) Each histogram shows the percentage contribution of high positive, positive, low positive, and negatively stained cells, as well as the final score of each protein expression. IHC: immunohistochemical; BCL-2: B-cell lymphoma 2; BAX: BCL-2-associated X protein; MTX: methotrexate.

To evaluate the possible protective effects of crocin against MTX-induced hepatotoxicity, IHC labeling was used to examine the expression of pro-apoptotic protein *BAX*, anti-apoptotic protein BCL-2, and caspase-3 (apoptosis coordinating enzyme) in liver tissues from various research groups. Both the untreated control group and the crocin-treated group showed low positive *BAX* expression, with only a few hepatocytes demonstrating strong positive cytoplasmic immunostaining (Figures 4A, 4B, 5A, 5B). MTX administration increased the cytoplasmic expression of the pro-apoptotic marker *BAX*, with almost all hepatocytes showing strong *BAX* immunostaining (Figures 4C, 5C). *BAX* immunostained sections from crocin/MTX-treated groups revealed a marked decrease in *BAX* expression in the majority of hepatocytes (Figures 4D, 5D). Similarly, caspase-3 immunostaining represented low positive and positive expression in normal and crocin-treated groups, respectively (Figures 4E, 4F, 5E, 5F). MTX caused marked upregulation of caspase-3 immunostaining, which was detected in the cytoplasm and nuclei or hepatocytes (Figures 4G, 5G). This upregulated expression of caspase-3 detected in MTX-intoxicated rats was almost normalized after concomitant administration of crocin and MTX (Figures 4H, 5H).

On the other hand, the IHC assessment of anti-apoptotic marker *BCL-2* revealed considerable expression in both normal untreated and crocin-treated groups (Figures 4I, 4J, 5I, 5J). The level of *BCL-2* protein expression was moderately reduced in liver tissues of the MTX-treated group (Figures 4K, 5K). Concomitant administration of crocin and MTX significantly increased *BCL-2* expression to levels equivalent to the normal control group (Figures 4L, 5L). Hence, the current study's findings indicate that one potential mechanism of crocin's hepatoprotective activities against MTX-induced hepatotoxicity is through its action on *BAX*, caspase-3, and *BCL-2*.

## Discussion

MTX is a primary medication for treating some diseases such as specific subtypes of leukemia and lymphoma; however, its administration is accompanied by several adverse reactions that restrict its clinical application. Hepatotoxicity is one of the most prevalent and harmful adverse reactions of MTX. Numerous studies have demonstrated that MTX and its metabolites induce inflammatory processes, oxidative stress, fibrosis, and apoptosis in hepatocytes [17,21]. The primary aim of the present study was to determine whether crocin might attenuate the cytotoxic effect of MTX on liver cells. To reach our objective, we evaluated the liver function, oxidative stress, inflammatory marker, *TGF-β1* (the master regulator of fibrosis), apoptosis, and histological alterations in liver tissue.

In this experiment, a single injection of MTX (20 mg/kg) resulted in a substantial increase in the levels of hepatic enzymes, including ALT, AST, and ALP, when compared to the normal control group (p < 0.001), which is consistent with the findings of preceding research [6,22,23]. Furthermore, crocin administration in combination with MTX considerably decreased the serum levels of three assessed liver enzymes compared to the MTX-treated group; however, except for the AST level, none of these differences were statistically significant. These findings were partially consistent with those of Akbari et al., who demonstrated that crocin pretreatment of rats with ischemia-reperfusion hepatic injury lowered the level of liver enzymes in blood [24]. The effect of crocin on liver function tests can be justified by its membrane-stabilizing activities that prevent the intracellular enzymes from leakage into the blood [22]. Furthermore, another study reported the effect of carotenoids, which include crocin in enhanced liver regeneration and repair, and consequently the liver aminase enzyme levels return to normal levels [25].

Concerning the oxidative stress markers, our finding revealed a significantly higher level of MDA and a significantly lower level of GSH as well as reduced enzymatic activity of SOD and CAT compared to the normal untreated group and the crocin-treated group (p < 0.001). Our results confirm the oxidative stress induced by the administration of MTX in hepatic tissues reported in previous studies, which reported an increase in MDA and reactive oxygen metabolites and a decrease in GSH, SOD, and glutathione peroxidase (GP-x) in MTX-treated rats when compared with the control group [26,27]. In agreement with two previous studies [14,28], the results of the current study indicated that pretreatment with crocin combined with MTX significantly increases the GSH level in liver homogenate, as well as enhances the activity of fundamental antioxidant enzymes (SOD and CAT), while substantially lowering MDA level in hepatic tissue when compared with MTX-treated group. In light of this, the observed effectiveness of crocin to counteract the hepatotoxic effects of MTX can be attributed to an increase in GSH synthesis, enhancement of SOD and CAT enzymatic activity, and a decrease in reactive oxygen species production (MDA).

Our results from biochemical analysis demonstrated a non-significant decrease in the level of CRP in both MTX-treated and crocin/MTX-treated groups when compared with normal control. This finding can be explained by the well-known anti-inflammatory and immunosuppressant effects of MTX [29,30]. In addition, these data are supported by the histological analysis of H&E-stained sections, which revealed no inflammatory reaction associated with the detected necrosis of hepatocytes in the MTX-treated group. This can be attributed to the fact that necrotic cell death, which occurs first, stimulates inflammatory responses, which might take a longer time to be detected [31]. However, the MTX dose administered in the current study has been reported previously to induce marked elevation of pro-inflammatory cytokines such as interleukin 6, interleukin 12, and TNF-α [17], which is contradicting our results. This contradiction can be justified by the difference in the time between MTX administration and rat scarification in both experiments. Further research is needed to resolve this issue and to better understand how MTX and crocin influence the inflammatory process.

In the present study, the expression of *TGF-β1* mRNA was increased in MTX-treated rats, although no fibrosis was detected in the histological study of liver tissue from the same group. *TGF-β1*, a cytokine that promotes fibrosis, enhances collagen synthesis and deposition of extracellular matrix and can stimulate the transformation of hepatic stellate cells to myofibroblast [32]. It has been reported that *TGF-β1* regulates the mitogen-activated protein kinase (MAPK) signaling and suppressor of mothers against decapentaplegic homolog 2/3 (*SMAD2/3*) pathways to drive stellate cell activation [33]. Additionally, inhibiting the *TGF-β1* signaling pathway can possibly interrupt the advancement of liver disease [34]. In our study, we detected that crocin's anti-fibrotic effects might be attributed to its ameliorative action on *TGF-β1*; hence, crocin helps to protect the liver against MTX-induced hepatotoxicity. These findings were in line with that of Algandaby [35], who discovered that crocin reduced *TGF-β1* expression, which in turn prevented collagen deposition in the treated group's liver.

For more confirmation of our biochemical findings, we studied the putative protective action of crocin on the liver by undertaking extensive histopathological and immunochemical investigations. Compared to the negative control group, administration of MTX resulted in a significant distortion of the histological architecture of the hepatic tissue. Hepatocytes showed hydropic degeneration with variable stages of necrosis, as well as whole areas of cellular absence were detected with variable-sized areas of necrotic foci. Alternatively, pretreatment with crocin along with MTX improved liver architecture when compared to the MTX-treated group, which is consistent with Chhimwal et al. who demonstrated the protective effect of crocin on the histological structure of liver [36].

We further assessed the effect of crocin on hepatocyte apoptosis by assessing *BAX* (pro-apoptotic), *BCL-2* (anti-apoptotic), and caspase-3 (apoptosis coordinating enzyme) protein expression in hepatic tissue of different study groups using IHC. Our results revealed that MTX administration enhanced the expression of the proteins *BAX* and caspase-3 while decreasing the expression of *BCL-2*, which led to an increase in hepatocyte apoptosis. However, the current study's findings indicated that concurrent administration of crocin and MTX reversed MTX-induced hepatotoxic effects by decreasing *BAX* and caspase-3 on one hand while increasing the expression of the *BCL-2* protein on the other. These IHC findings were in agreement with several previous studies [22,28]. As a result, the current IHC data suggest that one possible mechanism of crocin's hepatoprotective actions against MTX-induced hepatotoxicity is through its influence on *BAX*, caspase-3, and *BCL-2*. Taken together, our findings strongly emphasize that crocin protects the liver from the damage caused by MTX, which can be attributed in part to its potent antioxidant and anti-fibrotic actions.

One important limitation of the current study is the short duration of crocin administration, whereas long-term use of crocin therapy may have had a more effective protective effect. Similarly, a single dose of MTX has been assessed; however, MTX is usually used as a part of regimens to treat chronic diseases. Though further research should concentrate on using different doses of MTX for longer durations. In addition, due to a limitation of resources, we were unable to investigate further the influence of MTX and crocin on the inflammatory process. More investigations are needed to validate our results and to further understand how crocin affects other molecular mechanisms implicated by MTX administration in the liver.

## Conclusions

In conclusion, our findings confirmed the hepatotoxic effect of MTX as its administration induced deterioration of liver enzymes (AST, ALT, and ALP), an increase in oxidative stress damage, lipid peroxidation, and apoptosis in the liver, as well as an increase in *TGF-β1* expression. Significantly, the results of the current study demonstrated how crocin protects against MTX-induced hepatotoxicity. Our findings indicate that this effect is a result of the compound's antioxidant (reduced MDA and elevated GSH, CAT, and SOD), anti-apoptotic (upregulation of *BCL-2* and downregulation of caspase-3 and *BAX*), and anti-fibrotic (downregulation of *TGF-β1* gene expression) properties of crocin, as well as its ability to regulate the liver enzymes AST, ALT, and ALP. Furthermore, crocin treatment also restored the normal histological architecture of liver tissue in rats treated with MTX. As a result, the findings presented in this work utilizing an in vivo animal model support the idea that crocin should be explored further in humans to evaluate its putative hepatoprotective effects against MTX-induced hepatotoxicity.
